# The footprint of ship anchoring on the seafloor

**DOI:** 10.1038/s41598-022-11627-5

**Published:** 2022-05-07

**Authors:** Sally J. Watson, Marta Ribó, Sarah Seabrook, Lorna J. Strachan, Rachel Hale, Geoffroy Lamarche

**Affiliations:** 1 National Institute of Water & Atmospheric Research (NIWA), Auckland, New Zealand; 2 Institute of Marine Science, The University of Auckland, Auckland, New Zealand; 3 School of Science, Department of Environmental Science, Auckland University of Technology, Auckland, New Zealand; 4 School of Environment, The University of Auckland, Auckland, New Zealand

**Keywords:** Environmental impact, Geomorphology, Geophysics

## Abstract

With the COVID-19 pandemic came what media has deemed the “port congestion pandemic”. Intensified by the pandemic, the commonplace anchoring of high-tonnage ships causes a substantial geomorphologial footprint on the seabed outside marine ports globally, but isn’t yet quantified. We present the first characterisation of the footprint and extent of anchoring in a low congestion port in New Zealand-Aotearoa, demonstrating that high-tonnage ship anchors excavate the seabed by up to 80 cm, with the impacts preserved for at least 4 years. The calcuated volume of sediment displaced by one high-tonnage ship (> 9000 Gross Tonnage) on anchor can reach 2800 m^3^. Scaled-up globally, this provides the first estimates of the footprint of anchoring to the coastal seabed, worldwide. Seafloor damage due to anchoring has far-reaching implications for already stressed marine ecosystems and carbon cycling. As seaborne trade is projected to quadruple by 2050, the poorly constrained impacts of anchoring must be considered to avoid irreversible damage to marine habitats.

## Introduction

Since the beginning of the COVID-19 pandemic, thousands of ships have been reported waiting on anchor outside heavily congested ports^1–5^. Marine ports around the world have been experiencing unprecedented bottlenecks in traffic, with no relief in sight^6,7^. While the economic fallout of the pandemic on the global shipping industry is well reported^1,8–10^, the associated environmental impacts due to intensifying anchorage use have been little considered. The short-term deployment of anchors has been referred to as a “hidden cost” of the shipping industry^11^ due to the associated, and mostly unaccounted for, seabed damage^11,12^. The global pandemic has shone a spotlight on surging marine port congestion^1,13,14^. Concomitant anchorage use is becoming a more dominant, but unreported and unquantified, impact of the shipping industry on the global seabed^15^.

Physical damage to the seabed by ship anchors is increasingly considered a threat to the health of benthic communities^11,12,16–23^, due to physical destruction and associated changes in sediment type and ecosystem function^24^. The physical footprint of anchoring is likened to that of bottom trawling, the most widespread human impact on global continental shelves^20,25,26^. Bottom trawling can modify seafloor topography^22^, destroy benthic habitats^20,27^ and modify ecosystem processes^28,29^. Like bottom trawling, the ecological and biological impacts of anchoring is a function of the footprint of equipment type used, the seabed substrate^28,30^, the frequency of anchoring practices and the ecosystem resilience^26,31–33^. Although anchoring practices are limited to a narrower and shallower depth range (10–80 m water depth) than most bottom trawlers, they occur more frequently and more intensely (deeper seabed penetration). The impact of anchoring may be an unreported but significant contributor to the environmental footprint of the shipping industry which already includes the spread of invasive species, the production of greenhouse gas emissions, as well as air, water, and noise pollution^11,34^.

Our ability to quantify the extent of human activities modifying the seafloor and therefore measure the severity of the impact to marine ecosystems is limited by a paucity of high-resolution bathymetry data^11,35,36^. As such, the physical footprint and spatial extent of anchoring in water depths greater than 10 m remains elusive^11^, particularly for high-tonnage ships (> 9000 Gross Tonnage; GT), which have much larger, more destructive anchoring gear compared to recreational vessels.

To date, the environmental footprint of anchoring is not considered in global compilations of human impacts in marine ecosystems^25,37,38^, nor does data exist to evaluate the release of CO_2_ that may result from anchoring practices. As nations work to meet climate goals outlined in COP26 proceedings, a shift away from high-emission transportation, such as air freight^39^, is being encouraged. In November 2021, the Clydebank Declaration for green shipping corridors was agreed upon^40^, which will see countries working towards a net-zero goal for global maritime shipping. Yet to achieve more sustainable and lower-impact shipping corridors, the hidden costs of ship anchoring must be incorporated into future global trade strategies^15^.

## Results

**Figure 1:**
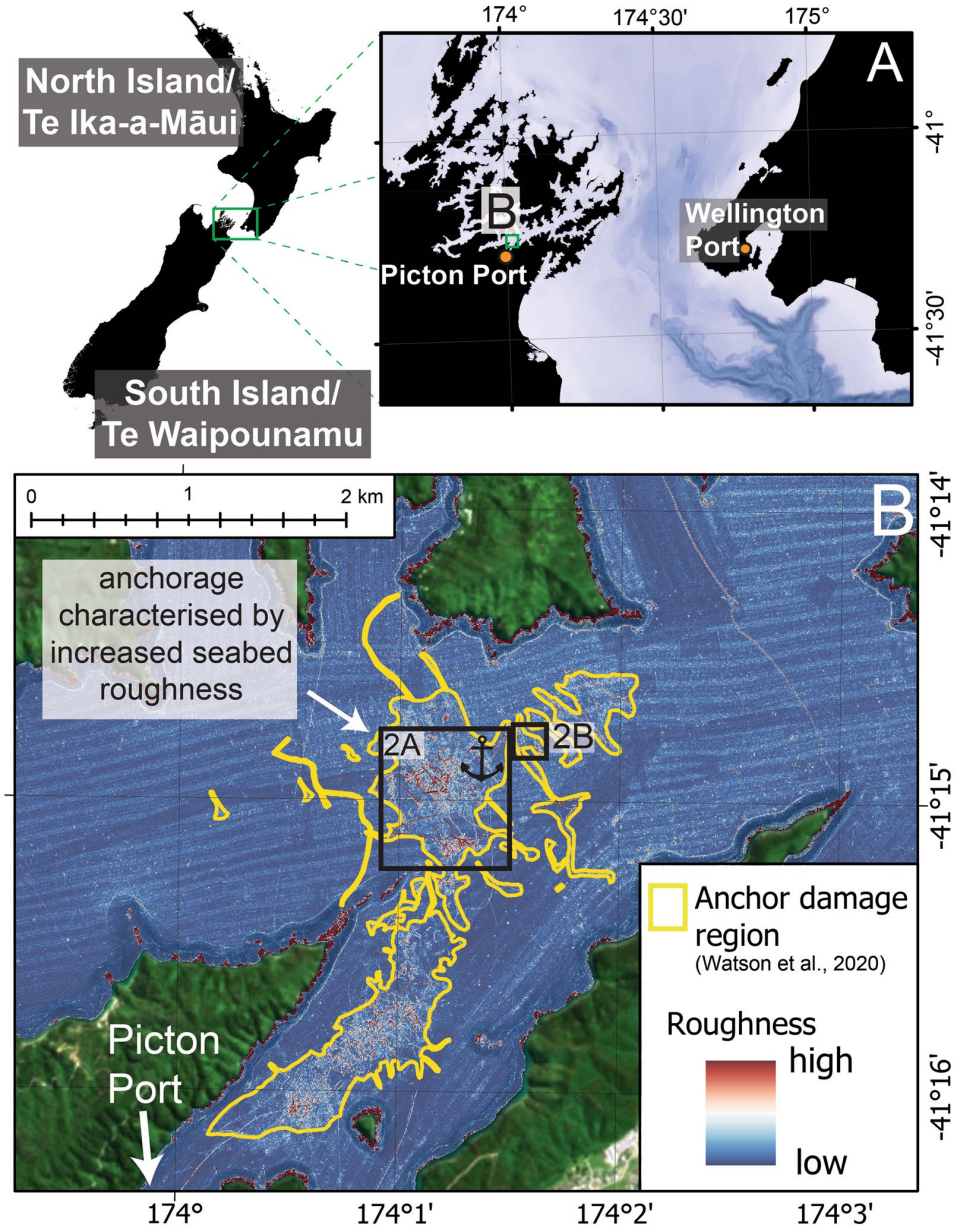
(**A**) Regional setting of the Picton Port case study, black polygons are landmasses. (**B**) Map of the Picton region showing region characterised by increased seafloor roughness due to anchoring practices (yellow polygon)^42^. Locations for Fig. 2A,B are shown by black boxes. Background satellite image obtained from Land Information New Zealand LINZ, 10 m (2018–2019). All figures were constructed using ArcGIS PRO version 2.8.3 and Adobe Illustrator 2022.

**Figure 2:**
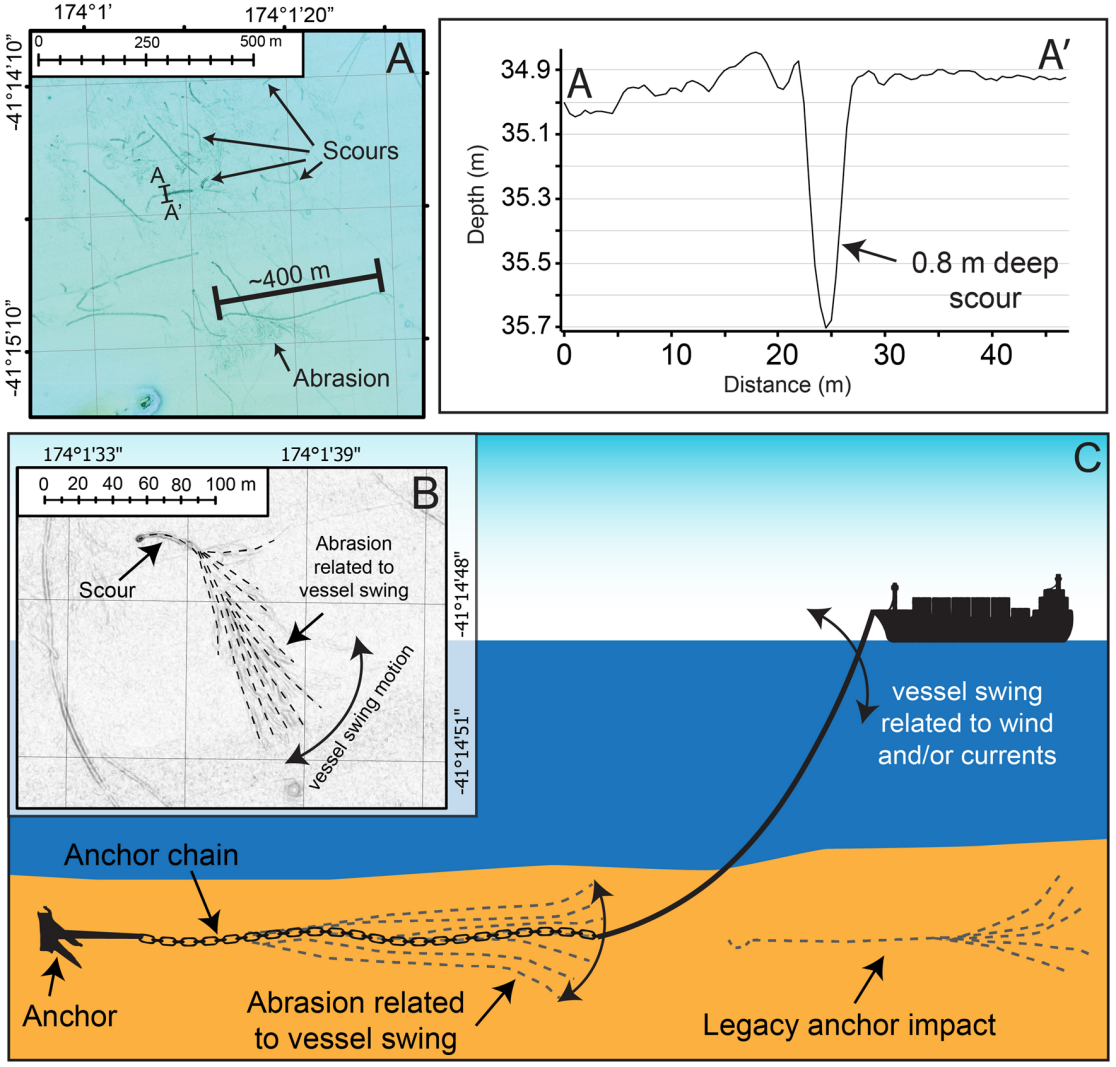
(**A**) Zoomed in bathymetry of the seafloor within the Picton anchorage (for location of figure see Fig. 1B). Scours and abrasion zones are labelled and the location of Profile A-A’ is annotated. Profile A-A’ shows the bathymetric profile where the penetration of one scour on the seabed is observed, of up to 5 m wide and 80 cm deep. (**B**) Shaded relief image of the seafloor showing a “broomstick-like” feature (interpretation in black dashed-line), reflecting the preservation of the anchoring gear impacting the seabed, and abrasion marks relating to the movement of the vessel (and chain scope) whilst on anchor. (**C**) Schematic representation of how anchoring gear (including the anchor and the chain scope) impact the seabed leaving scours, abrasion zones and “broomstick-like” features.

Multibeam bathymetry data in the vicinity of the small Picton anchorage, South Island New Zealand, reveals the footprint of anchoring, characterised by increased seafloor roughness (Fig. 1A,B). The geomorphological expression of anchoring is mostly concentrated at ~ 35 m water depth and extends from the designated anchorage site, more than 3 km south towards the Picton Port (Fig. 1B). There are two main morphological signatures within the anchorage region: (1) linear scours, attributed to the anchor impacting the seabed, either during anchor emplacement or recovery, and (2) zones of abrasion characterised by irregular seabed roughness, attributed to anchor chain movement and swing while the ship is on anchor (Fig. 2A,B). Individual scours observed at the Picton anchorage are regularly over 400 m in length, ~ 5 m wide and range from 40 to 80 cm deep (Fig. 2A, Profile A-A’). Linear scours and abrasion zones are often found adjacent to each other, forming “broomstick-like” features on the seafloor, reflecting where anchor emplacement (scour) and ship swing (abrasion) has been preserved on the seabed (Fig. 2B,C).

**Figure 3:**
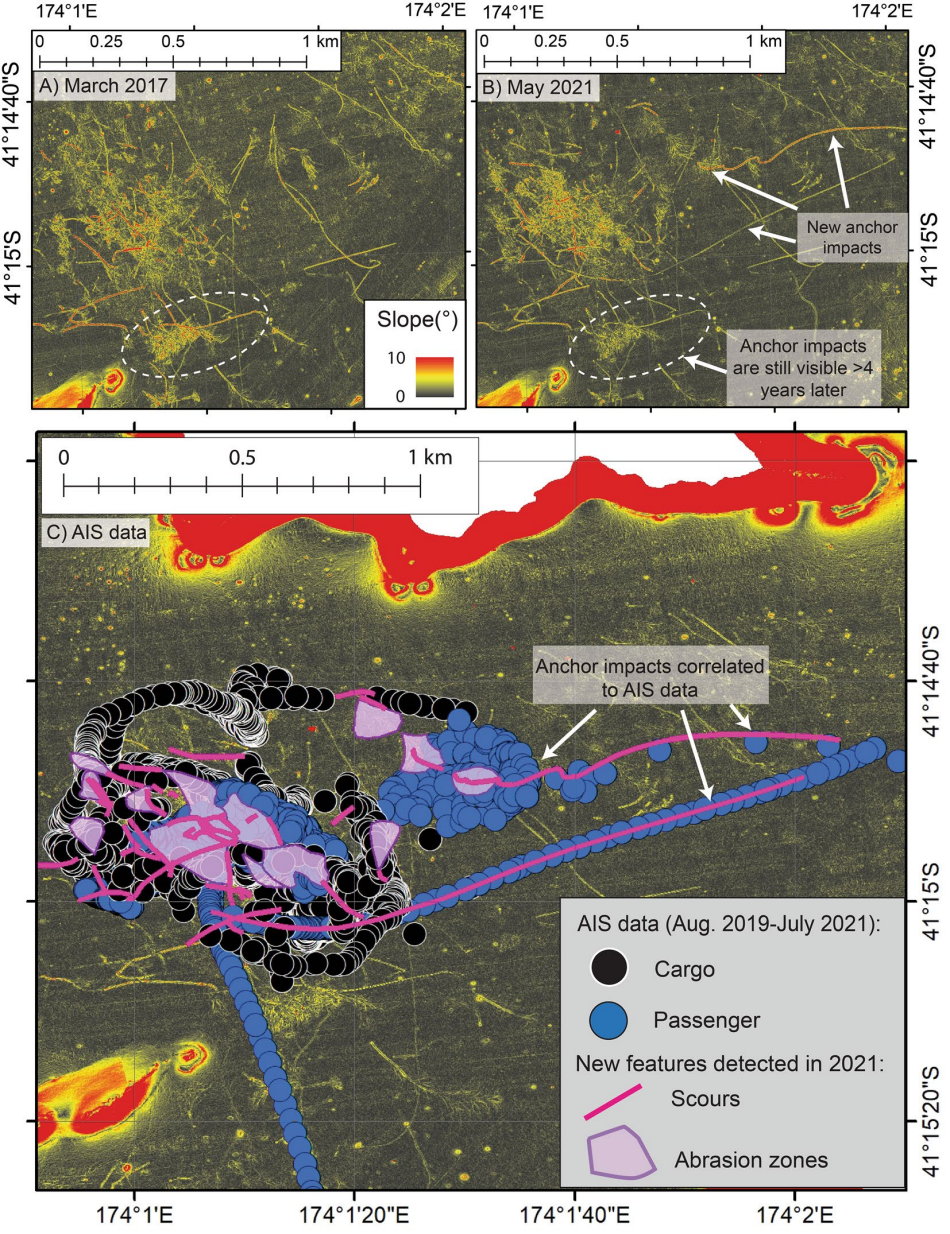
Seafloor slope showing a snapshot of the anchor footprint in Picton in 2017 (**A**) and in 2021 (**B**). The 2021 survey reveals new scours and abrasion zones that have occurred since the 2017 survey. White dashed circle shows the same anchor marks are visible in both datasets suggesting that the physical footprint of anchoring persists on the seabed for at least 4 years in mud-dominated substrate, although with more subdued appearance. (**C**) Seafloor slope with new scours (pink lines) and abrasion zones (purple polygons) that were digitised in 2021 survey. AIS ship tracking data over 2-years (August 2019-2021) shows that high-tonnage cargo (black points) and passenger (blue points) vessels use the Picton anchorage during this time. In some cases, AIS data can be directly correlated to individual anchor impacts on the seabed (example labelled).

We use two repeat bathymetric surveys of the Picton anchorage (collected in 2017 and 2021) and ship tracking data (herein referred to as AIS data) to estimate the area and volume of sediment disturbance by anchoring practices (Table 1), and link physical footprints on the seafloor to anchoring by specific ships. At Picton anchorage, the depth of seabed penetration by anchoring gear is up to 80 cm and the total areal extent of the anchor damage region is 1.8 km^2^ (Table 1). From these observations we estimate that the total volume of sediment disturbed by anchoring at Picton is 1.4 million m^3^ (Table 1). The area impacted by anchors in the four years between the two bathymetry surveys is 0.1 km^2^, and up to 98,400 m^3^ of seabed sediment displaced (Fig. 3A–C; Table 1). The anchorage region in Picton that was mapped in the initial bathymetry dataset (2017), is still observable in the repeat survey (2021; Fig. 3A,B). This represents an anchoring footprint that is preserved over more than 4 years in a low energy environment, where the seafloor is composed of muddy substrate (92.8% mud)^41,42^.

**Table 1:** Calculations of seabed area and volume disturbed by anchors based on observations from Picton and Wellington anchorages. See methods and Supplementary Table 1 for detailed calucations.

	Area (m^2^)	Maximum volume (m^3^)
Estimated footprint for one ship on anchor	3416	2733
Documented footprint over 4 years (2017–21) at Picton	123,000	98,400
Total footprint observed at Picton, NZ	1.8 × 10^6^	1,440,000
Total footprint observed at Wellington, NZ	6.2 × 10^6^	4,960,000
Estimated global footprint (in < 80 m water depth)	5971–20,565 × 10^6^	5–16 × 10^9^

AIS data shows there were 18 occasions when ships were recorded on anchor at Picton between August 2019 and August 2021. The ships using the Picton anchorage are high-tonnage passenger (n = 9) and cargo (n = 9) ships (Fig. 3C). Using the total number of ships on anchor provided by AIS data (n = 18) and the additional anchor footprint mapped over the two bathymetry surveys (0.1 km^2^) we provide the first estimates of the average spatial impact and sediment volume of one individual high-tonnage ship on anchor (Table 1). We calculate that each time one high-tonnage ship is on anchor on average 3,416 m^2^ of seabed area is impacted. Assuming 80 cm anchor gear penetration, this equates to 2,733 m^3^ of seabed sediment displaced (Table 1), more than enough sediment to fill an Olypmic-sized swimming pool every time a ship anchors. However, using both AIS and repeat bathymetry data, a footprint covering 24,500 m^2^ of seabed can be correlated to one 134 m ship with on anchor status for 15 h in April 2020 (Fig. 3C). This is seven times the footprint determined in our average estimations and demonstrates that individual ships on anchor are capable of producing a much larger spatial footprint than average estimates may conclude.

In Picton, the average time high-tonnage ships spent on anchor between August 2019 and 2021 was 0.8 days, with cargo ships spending more time on anchor compared to passenger ships (average 1.3 and 0.4 days respectively). The time spent on anchor ranged from 30 min to 3.4 days, and the average ship size was 170 m long and ~ 23,000 GT. In 2020, 14 ships were documented on anchor, 2 in the last 5 months of 2019 and 2 in first 8 months of 2021. For Picton, anchorage use in 2020 likely represents an overestimate of typical yearly anchorage use, as 2020 is a year known for anomalously high port congestion globally^1^.

## Discussion

**Figure 4:**
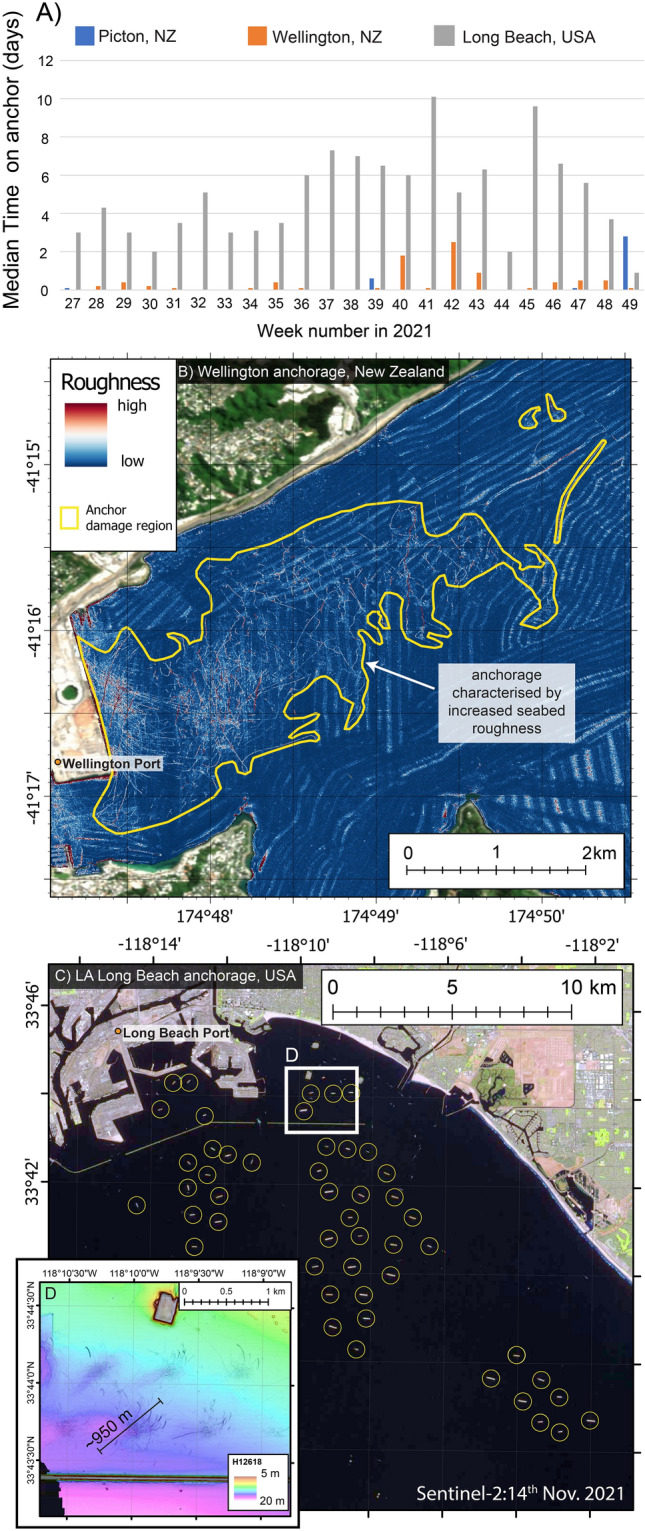
(**A**) Documented anchorage use for 23 weeks in 2021 at Picton anchorage, NZ, compared to Wellington anchorage, NZ, and Long Beach anchorage, USA. Data obtained from Marine Traffic (https://www.marinetraffic.com). The median anchorage use per week in Picton is substantially lower than all other ports presented and likely represents a low-congestion anchorage area globally. (**B**) Seafloor data from Wellington anchorage region (collected in 2007–2008^44^) showing region characterised by increased seafloor roughness in the anchoring region (yellow polygon). Background satellite image obtained from Land Information New Zealand LINZ, 10 m (2018–2019). (**C**) Sentinel-2 Satellite imagery data (obtained from https://earthexplorer.usgs.gov/ Entity ID: L1C_T11SLT_A024506_20211114T184314, Tile number: T11SLT) showing at least 49 ships on anchor outside the LA Long Beach port on the 14th November 2021. This likely represents the spatial expansion of the anchorage region as a result of unrelenting port congestion since the beginning of the COVID-19 pandemic and an example of the potential future of port congestion. (**D**) Bathymetry data (collected in 2013^45^) from Long Beach anchorage showing anchor footprint analogous to that observed at Picton.

Data presented in this study shows that the seabed footprint due to anchoring practices at Picton anchorage is extensive and is preserved for more than 4 years. This is the first time repeat bathymetry surveys over anchorage sites have been published allowing us to constrain the longevity and characterise the full extent of the physical impacts caused by high-tonnage ships on anchor (Fig. 3A–C). Of the 3669 martime ports globally, 3317 have documented information about anchorage sites for ships awaiting port calls^43^. Based on the spatial extent of the anchoring footprint at Picton, we estimate that the absolute minimum area of seabed impacted by high-tonnage ship anchoring globally is at least 6,000 km^2^ (Table 1). This modest global footprint is necessarily a substantial underestimate as Picton is a low congestion port. In higher congestion ports in New Zealand, such as Wellington, and heavily congested ports worldwide, such as Long Beach, USA, anchoring occurs more frequently, for longer durations (Fig. 4A). In more congested ports, the anchoring footprint accordingly extends over a much wider region. For example, the anchoring footprint near Wellington Port extends over 6.2 km^2^ (Fig. 4B), which if extrapolated globally equates to more than 20,000 km^2^ of shallow coastal seabed impacted by anchors (Table 1). Bathymetry data collected prior to the global pandemic from outside both Wellington (2007–2008)^44^ and Long Beach (2013)^45^ ports shows the characteristic anchor footprint (Fig. 4B,D), analogous to that observed at Picton anchorage. We do not have repeat bathymetry surveys of Wellington or Long Beach anchorages collected since the pandemic. However, satellite imagery data collected from Sentinel-2 in November 2021 shows at least 49 ships on anchor outside Long Beach Port, encompassing an area greater than 90 km^2^ (Fig. 4C)^46^. This indicates that the area for high-tonnage ships to anchor at Long Beach Port has expanded since 2013 due to increasing port congestion. As the reliance on seaborne trade and port congestion intensifies worldwide^1,47–50^ the seabed area that is being impacted will increase from outside of classic anchorage zones to the wider coastal environment. One of the major impacts of intensifying port congestion has been increased time at anchor^1,51^. Our results show that the combination of anchor emplacement and time spent on anchor results in a larger spatial footprint on the seabed. Picton Port has relatively low congestion in the global context and therefore has correspondingly low anchorage use, both in terms of the number of ships using the anchorage and the amount of time individual ships spend on anchor (Fig. 4A). This is reflected by the anchor footprint nearby Picton anchor site (1.8 km^2^) being three times smaller than that of Wellington Port (Fig. 4B), and 50 times smaller than the estimated region impacted by ship anchors at Long Beach anchorage (Fig. 4C). In general, high-tonnage ships entering Picton Port will make immediate port calls (i.e., no need for anchorage use), and if they do use the anchorage, the median use time is up to 0.1 days (Fig. 4A). For comparison, Wellington Port has more frequent and longer duration anchorage use (up to 0.4 days on anchor median per week; Fig. 4A). Similarly, reports in newsmedia highlight substantial increases in the number of ships reported on anchor outside the Long Beach port since 2020^52^, as well as increases in the time spent on anchor (in some cases > 7 days; Fig. 4A). The scale of seabed impact attributed to anchoring at the low congestion Picton anchorage would likely be more widespread at more congested ports around the world, due to more frequent and longer duration anchoring. As such, our results from Picton represent the minimum expected anchor footprint in coastal environments.

Our estimates of the global anchoring footprint of between 6000–20,565 km^2^ (Table 1) is much less than the 9.74 million km^2^ of continental shelves impacted by fisheries trawling^53^. Yet, our results indicate that anchoring is a major global cause of seabed and benthic habitat degradation for three reasons: (1) impacts from ship anchoring is restricted to a narrower and shallower water depths, (2) anchoring gear results in consistently deeper seabed penetration than trawling gear, and (3) anchoring occurs more frequently across the same regions compared to the frequency of bottom trawling spanning over more extensive areas.

Compared to bottom trawling, which occurs across a wider range of water depths^22,26,53^, anchoring practices for high-tonnage ships are mostly concentrated in shallow waters between 10 and 80 m^11,54,55^. For example, along the French coast of the Mediterranea Sea, anchoring of high-tonnage and recreational ships is estimated to have adversely impacted ~ 30% of habitats between 0–80 m^54^. In fact, anchoring in water depths greater than 80 m is considered “deep water anchoring” and is not recommended by the International Association of Independent Tanker Owners^55^, whereas trawling depths typically span 0–1500 m^22,26,53^.

Modelling of anchor impacts suggests that anchoring gear can regularly excavate 1 m into the seabed, with larger anchors more likely to cause deeper excavation^56^. Bathymetry data presented in this study confirm these modelling results (Fig. 2A), and demonstrate that anchoring excavates the seabed at scales comparable to bottom trawling gear^11,12^. The penetration depth of trawling gear in soft sediment substrates ranges from a few centimetres (Otter Trawl) to ~ 16 cm (Hydraulic dredge), with the associated adverse impacts on benthos increasing with the penetration depth of the trawling equipment^18–20^. Use of Otter Trawls and Scallop Dredges has been demonstrated to change benthic sediment organic carbon content and biogeochemical cycling^28^. Bottom trawling impacts have been shown to reduce invertebrate abundance by 26% and species richness by 19%, and change benthic species composition (when disturbed by gear with penetration depth greater than 16 cm)^18–20^. In these instances, recovery of benthic community abundance does not occur within 3 years of seabed disturbance^20^.

The maximum seabed penetration by anchoring gear observed within the Picton anchorage site is 80 cm (Fig. 2A), approximately five times the penetration depth of most benthic trawling equipment. The upper 1 m of the seabed stores up to twice as much carbon of terrestrial soils, being highly effective in global carbon sequestration ratio^57^. As such, areas that are impacted by anchoring practices need to be included when calculating global marine carbon stocks^58^.

Anchor impacts have also been observed to last on the seafloor over decadal timescales^59^, although recovery of the seabed and associated habitats varies with species and seabed substrate^19,60,61^. The anchor footprint in Picton that is visible in both bathymetry datasets, presenting as more subdued in the latter survey (~ 4 years later) demonstrated by diminished slope (surface roughness) signature (Fig. 3A,B). Although this indicates the beginnings of physical recovery of the seafloor, this is not a proxy for recovery of the habitat itself.

Periodic disturbance by anchors of all ship types and sizes (from recreational to cargo) has been shown to adversely affects all habitat types (e.g., rocky reef or soft sediment)^12,62,63^. Seabed habitat recovery from benthic trawling is estimated to be faster for coarse-sediment (sand) regions compared to fine-sediment regions (mud)^19,60,61^, the latter being the preferred substrate for anchoring^64^. Studies suggest that bottom trawling frequencies of just once per year will result in average 15.5% decrease in benthic biomass, with recovery estimated to be > 3 years^19^. Intensively trawled areas are those that are trawled between 1–10 times per year^19^. For comparison, at the Picton anchorage, we observe 18 occasions where high-tonnage ships were “on anchor” over ~ 2 years. Although these values are likely to be inflated due to COVID-19 related port congestion, other ports globally exhibit more frequent anchoring both before and after COVID-19. For example, at the port of Gothenburg, Sweden, there were 55 occasions where cargo ships were documented to be on anchor in one month (August, 2014)^65^. For European ports in the Baltic Sea, there was nearly 15,000 occasions estimated where ships were anchored for one year (2015)^66^. Satellite images of the Long Beach Port provide a snapshot that reveals at least 49 ships on anchor in November 2021 (Fig. 4C), with consistently high frequency and long duration anchorage use reported for the entire of 2021 (Fig. 4A)^52^.

We suggest that the concentrated, and high frequency seabed excavation due to high-tonnage ship anchoring within shallow waters (< 80 m) likely has a range of physical, biological and chemical consequences^67^. These may include, but are not limited to those associated with any disturbance to the seabed by human equipment, such as pollution by heavy metals (from bottom contact gear), loss of surface sediment, modifications to modal grainsize, increasing sediment resuspension, removal and/or changes in faunal populations^68,69^, denitrification, and changes to biogeochemical cycling^23,70^. As such, the benthic communities disturbed by anchoring gear likely experience similar, if not more intense impacts than those caused by bottom trawling gear.

According to the United Nations Review on Maritime Transport^71^, the future of global trade will have an increased reliance on shipping^47^. Shifts towards greener forms of transport to reduce carbon emissions will likely involve increasing reliance on seaborne methods for trade, due to the relatively low carbon emissions compared to airfreight^72^. The COP26 Clydebank Declaration^40^ (signed on the 10th of November 2021) represents the initial stages in global planning for more sustainable and lower impact international shipping corridors. However, anchoring impacts have yet to be recognised within these frameworks.

In 2020, the COVID-19 pandemic put the global shipping industry on hold due to border restrictions and reduced human mobility^73^. As global marine traffic halted, port congestion skyrocketed due to limited storage capacity within ports resulting in intensive anchorage use reported around the world^1,8^. More than a year since the beginning of the COVID-19 pandemic, restrictions are easing globally yet the “port congestion pandemic” prevails. The increasing rates of marine port congestion and associated anchorage use observed since the beginning of the pandemic represents an insight into the future of port congestion. With global marine traffic predicted to increase in the coming decades, it will be critical that we develop better strategies of managing high-tonnage ships awaiting port calls to meet the COP26 agreement and mitigate damage to sensitive shallow marine areas.

The COVID-19 pandemic has reshaped the global shipping industry, shining light on future issues relating to unrelenting port congestion. Until now, the extent, intensity, and persistence of seabed impacts by anchoring of high-tonnage vessels have remained largely out-of-sight. This study uncovers that the reliance on shipping for international trade and travel, and the commonplace use of anchorage sites for maritime operations, represents a major, yet unaccounted for, driver of shallow benthic habitat destruction. The global seabed footprint associated with these anchorage practices may exceed 20,000 km^2^ of seafloor. The future environmental sustainability of the shipping industry requires improved quantitative assessment of the impact of anchoring practices on seabed habitats worldwide so that robust mitigating approaches can be developed and implemented.

## Conclusions

Recent high-resolution mapping efforts outside the low congestion port of Picton, New Zealand has revealed that anchoring of high-tonnage vessels has an extensive and persistent physical impact on the seafloor. This is the first study to be published which documents the morphology and extent of anchoring outside a marine port. In this study, we present evidence that high-tonnage ship anchoring can excavate the seafloor up to 80 cm, and individual ships can displace 2,800 m^3^ of sediment. This physical disturbance would be enough to induce variations of sedimentation patterns, destroying soft sediment habitats and ecosystem function. Repeat multibeam bathymetry shows that the footprint of anchoring is preserved on the seabed for ~ 4 years. Globally, Picton anchorage has relatively low maritime congestion, suggesting that the impact of regular anchoring worldwide likely represent a major driver of shallow marine habitat degradation. Our findings are consistent with other research into the impacts of ship anchoring, indicating that new solutions are required to reduce the global impact of anchorage congestion on the seabed^11,12,66^. With the increasing trends in global marine traffic predicted in the coming decades, a less destructive method of managing high-tonnage vessels (e.g., waiting on port calls using the engine instead of anchoring or slowing vessel speed on approach to port^66^) is necessary to mitigate the global impact of maritime activities on the seabed.

## Methods

We use two high-resolution multibeam bathymetry datasets collected during two surveys run across the Picton anchorage (Fig. 1).

The initial survey consisted of a comprehensive mapping of the entire Queen Charlotte Sound including the Picton anchorage in March 2017^41,42^. The bathymetry data were collected by NIWA as a part of the Hydrographic Survey (HS51), commissioned by Land Information New Zealand (LINZ) and Marlborough District Council (MDC) within the Queen Charlotte Sound/Tōtaranui region^41,42^. This bathymetric dataset was collected to update nautical charts for safety of navigation and are certified by the Hydrographic Authority of New Zealand. Data collection followed strict LINZ HYSPEC standards and protocols: https://www.linz.govt.nz/sea/charts/standards-and-technical-specifications-for-our-chart-and-hydrographic-work. The survey was conducted, with a full Quality Assurance Data Pack and the primary survey is publicly available to view and download here: https://www.marlborough.govt.nz/environment/coastal/seabed-habitat-mapping/queen-charlotte-sound-totaranui-seabed-mapping. The second survey was conducted in May 2021, and targeted the Picton anchorage site to characterise the extent and persistence of anchor impacts across the ~ 4-year interval. Both surveys were conducted using NIWA’s RV *Ikatere*, with the 300 kHz dual head Kongsberg EM2040 mutibeam echosounder. Both datasets were gridded at 1 m resolution. Bathymetry data were also examined outside other marine ports (Wellington, New Zealand (1 m resolution)^44^, and Long Beach, USA (2 m resolution^45^) for morphological signatures of anchoring similar to those observed at Picton anchorage (Fig. 4B,C). The vessel tracking data system, also known as Automatic Identification System (AIS), provides real-time positioning of ship traffic, transmitted via VHF signals. In New Zealand, large (> 45 m) vessels are subject to the International Convention of Safety of Life at Sea (SOLAS), and must be equipped with AIS tracking capabilities. AIS data were supplied by MDC and include the position, speed, ship status and type of ship approximately every 6 min. AIS data were filtered spatially to include only the anchorage region, by speed (< 1 knot) and ship status (“on anchor”). Weekly modal anchoring information from other ports around the world was obtained from Marine Traffic (https://www.marinetraffic.com). Sentinel 2 satellite imagery data were derived from https://earthexplorer.usgs.gov/, Entity ID: L1C_T11SLT_A024506_20211114T184314, Tile number: T11SLT, collected on the 14th November, 2021. All bathymetry, AIS datasets and satellite imagery were examined in ArcGIS Pro (V2.8.3). Seabed roughness grids were created using Benthic Terrain Modeler V3.0^74^.

Estimates for the average spatial impact of individual ships on anchor were calculated using: (1) the total number of ships on anchor at Picton during a two-year interval obtained via AIS data and (2) the total difference in anchor footprint in seabed damage mapped in the 2021 bathymetry dataset, as compared to four-years earlier in the 2017 dataset. See Supplementary Table 1 for detailed calculations and values. The total anchor footprint calculated for the four years between surveys was divided by two, to account for the duration of available AIS data (two years), then divided by the number of ships “on anchor” over the two-year duration (n = 18). The total global seabed impact due to anchoring was estimated using the total area impacted by anchors in the Picton anchorage (1.8 km^2^) and Wellington anchorage (6.2 km^2^) multiplied by the number of anchorage sites in the world (n = 3317) according to the World Port Index^43^.

## Supplementary Information

Supplementary Information.

## Data Availability

The HS51 multibeam dataset related to this article can be found at https://data-marlborough.opendata.arcgis.com/search?tags=Multibeam, an open-source online data repository hosted at the Marlborough District Council website, and visualised at: https://marlborough.maps.arcgis.com/apps/MapSeries/index.html?appid=155a89b0beb74035bd1c4c71f6f36646.
